# Development of standard clinical endpoints for use in dengue interventional trials: introduction and methodology

**DOI:** 10.1186/s12874-018-0601-z

**Published:** 2018-11-15

**Authors:** Thomas Jaenisch, Kim Hendrickx, Martin Erpicum, Liane Agulto, Kay M. Tomashek, Walla Dempsey, João Bosco Siqueira, Morgan A. Marks, Michael P. Fay, Catherine Laughlin, Maina L’Azou, Yee-Sin Leo, Federico Narvaez, Remy Teyssou, Stephen J. Thomas, Hasitha Tissera, Derek Wallace, Annelies Wilder-Smith, Duane J. Gubler, M. Cristina Cassetti

**Affiliations:** 1 Section Clinical Tropical Medicine, Department of Infectious Diseases, Heidelberg University Hospital, Heidelberg, Germany; 2 Postdoctoral Fellow of the Research Foundation - Flanders (FWO) and Research Associate of Spiral, Université de Liège, Liège, Belgium; 3 Mesydel, SPIRAL Research Center, Département de Science Politique, Université de Liège, Liège, Belgium; 4 Division of Microbiology and Infectious Diseases, National Institute of Allergy and Infectious Diseases, National Institutes of Health, Rockville, MD USA; 5 Federal University of Goias, Goiânia, Brazil; 6 Pharmacoepidemiology Department, Merck & Co., Inc., Kenilworth, New Jersey, USA; 7 Biostatistics Research Branch, Division of Clinical Research, National Institute of Allergy and Infectious Diseases, National Institutes of Health, Rockville, MD USA; 8 Global Epidemiology, Sanofi-Pasteur, Lyon, France; 9 Institute of Infectious Disease and Epidemiology, Tan Tock Seng Hospital, and National Centre for Infectious Diseases MOH, Singapore, Singapore; 10 Infectious Diseases Unit, National Pediatric Reference Hospital, Hospital Infantil Manuel de Jesús Rivera, Managua, Nicaragua; 11 Partnership for Dengue Control, Fondation Merieux, Lyon, France; 12 Unité de Virologie, Institut de Recherche Biomédicale des Armées, Brétigny-rur-Orge, France; 13 Division of Infectious Diseases, State University of New York, Upstate Medical University, Syracuse, NY USA; 14 National Dengue Control Unit, Ministry of Health, Colombo, Sri Lanka; 15 Takeda Pharmaceuticals International AG, Zurich, Switzerland; 16 Lee Kong Chian School of Medicine, Nayang Technological University, Singapore, Singapore; 17 Emerging Infectious Diseases Programme, Duke-NUS Medical School, Singapore, Singapore

**Keywords:** Dengue, Severe dengue, Endpoints, Standardization, Validation, DELPHI, Vaccine, Therapeutic, Intervention, Pathophysiology, Epidemiology

## Abstract

**Background:**

As increasing numbers of dengue vaccines and therapeutics are in clinical development, standardized consensus clinical endpoint definitions are urgently needed to assess the efficacy of different interventions with respect to disease severity. We aimed to convene dengue experts representing various sectors and dengue endemic areas to review the literature and propose clinical endpoint definitions for moderate and severe disease based on the framework provided by the WHO 2009 classification.

**Methods:**

The endpoints were first proposed and discussed in a structured expert consultation. After that, the Delphi method was carried out to assess the usefulness, validity and feasibility of the standardized clinical disease endpoints for interventional dengue research.

**Results:**

Most respondents (> 80%) agreed there is a need for both standardized clinical endpoints and operationalization of severe endpoints. Most respondents (67%) felt there is utility for moderate severity endpoints, but cited challenges in their development. Hospitalization as a moderate endpoint of disease severity or measure of public health impact was deemed to be useful by only 47% of respondents, but 89% felt it could bring about supplemental information if carefully contextualized according to data collection setting. Over half of the respondents favored alignment of the standard endpoints with the WHO guidelines (58%), but cautioned that the endpoints could have ramifications for public health practice. In terms of data granularity of the endpoints, there was a slight preference for a categorical vs numeric system (e.g. 1–10) (47% vs 34%), and 74% of respondents suggested validating the endpoints using large prospective data sets.

**Conclusion:**

The structured consensus-building process was successful taking into account the history of the debate around potential endpoints for severe dengue. There is clear support for the development of standardized endpoints for interventional clinical research and the need for subsequent validation with prospective data sets. Challenges include the complexity of developing moderate disease research endpoints for dengue.

## Background

An increasing number of dengue vaccines and therapeutics are currently in clinical development, and many vaccines are in advanced clinical development. The vaccine trials, to date, have been designed using World Health Organization (WHO) guidelines for determining efficacy in endemic countries [1, 2], primarily focusing on prevention of new infections. The WHO guidelines also state that secondary endpoints for vaccine efficacy should include the effect of vaccines on disease severity and on clinical presentations, including atypical cases.

While the primary vaccine efficacy endpoints have been well-defined, there are no published consensus clinical trial endpoints to measure disease severity. A recently licensed dengue vaccine had moderate efficacy at preventing virologically-confirmed dengue symptomatic disease but higher efficacy against severe disease [3]. Protection against severe forms of disease has consequently been added to the efficacy portfolio of dengue vaccines, and each developer has crafted their own definition of severe disease based on expert opinion and independent data monitoring committee (IDMC) consensus [1, 4]. This lack of standardization impedes our ability to compare results between dengue clinical trials, and ultimately evaluate potential products.

Although few clinical trials of therapeutic strategies to treat dengue patients have been conducted, several drug candidates are nearing evaluation in clinical trials. In addition to the direct antiviral activity, an assessment of the impact on disease severity will be critical for evaluation of therapeutic interventions.

The WHO 2009 dengue classification defines the severe end of the disease spectrum as i) dengue shock/respiratory distress with fluid accumulation (plasma leakage), ii) severe bleeding, or iii) severe organ dysfunction [5, 6]. This categorization was recognized as a platform for the development and the operationalization of standardized clinical severe disease endpoints. While many clinicians and public health officials welcomed the usefulness and practicability of this classification for patient management [7, 8], the need for development of clearly defined endpoints to measure dengue disease severity for intervention trials and pathogenesis studies has been highlighted by the research community [6, 9–12].

In 2015, experts from academia, international public health institutions, pharmaceutical industry, government and non-government organizations convened four times to define standardized endpoints of moderate and severe dengue disease to facilitate interventional clinical research. After the structured expert consultation, the Delphi methodology was employed to refine the proposed clinical endpoints and assess the usefulness, validity and feasibility of these endpoints for interventional dengue research.

Here, we present the processes that were used to define the endpoints, including the structured feedback from the respondents on this project, and the plans for their validation. The finalized definitions proposed for moderate and severe dengue clinical endpoints and a novel dengue illness index tool for measuring moderate disease are reported in two separate papers.

## Methods

### Structured expert consultation (4 workshops)

**Fig. 1:**
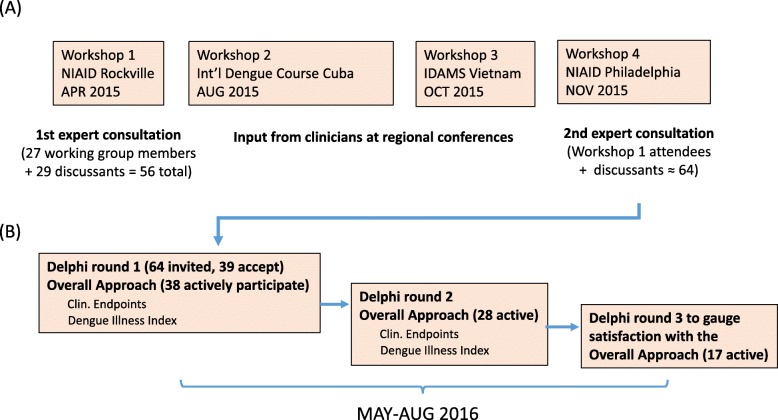
Workflow of workshops and Delphi. **a** Structured expert consultation (4 workshops). **b** The Delphi commenced with an invitation to participate in the OA panel followed by the option of participating in the panel for Clinical Endpoints or the Dengue Illness Index, or both

Structured expert consensus methods have been used successfully in Public Health, including the field of dengue research [8, 13]. In January, 2015, the National Institute of Allergy and Infectious Diseases (NIAID), part of the National Institutes of Health, and the Partnership for Dengue Control (PDC), convened a group of 27 experts to develop standardized clinical trial endpoints to measure dengue disease severity in interventional clinical trials (Workshop 1, see Fig. 1). The group included clinicians who follow and treat dengue patients, vaccine developers, academic researchers, and public health specialists from 14 different countries in Asia, the Americas and Europe (see Table 1). These dengue experts were identified via referral and selected to achieve a balanced representation of subject matter experts from all sectors and from various global endemic regions.

**Table 1:** Scientific working group members and their affiliating country

Clinical Endpoint Development			Dengue Illness Index	Endpoint Validation
Bleeding	Plasma Leakage	Severe Organ Involvement		
Lucy Lum^a^ (MYS)	Bridget Wills^a^	Laurent Thomas^a^ (MTQ)	Robert Edelman^a^	Thomas Jaenisch^a^
Norma de Bosch^a^ (VEN)	Elsa Rojas^a^(COL)	Kay Tomashek^a^	Stephen Thomas^a^	João Bosco Siqueira^a^
Alex Precioso (BRA)	Alexander Schmidt	Alex Precioso	Alex Precioso	Annelies Wilder-Smith (SGP)
Alexander Schmidt (BEL)	Beth Ann Coller	Anna Durbin (USA)	Alexander Schmidt	Beth Ann Coller
Beth Ann Coller (USA)	Cristina Cassetti	Cristina Cassetti	Catherine Laughlin (USA)	Catherine Laughlin
Bridget Wills (VNM)	Derek Wallace (SGP)	Duane Gubler	Cristina Cassetti	Cristina Cassetti
Cristina Cassetti (USA)	Emanuel Narvaez	Emanuel Narvaez	Emanuel Narvaez	Derek Wallace
Duane Gubler (SGP)	Hasitha Tissera (LKA)	Lucy Lum	Hasitha Tissera	Emanuel Narvaez
Emanuel Narvaez (NIC)	João Bosco Siqueira (BRA)	Piyarat Suntarattiwong	João Bosco Siqueira	Hasitha Tissera
Kay Tomashek (USA)	Kay Tomashek	Stephen Thomas (USA)	Kay Tomashek	Maina L’Azou (FRA)
Thomas Jaenisch (DEU)	Piyarat Suntarattiwong (THA)			Michael Fay (USA)
Yee-Sin Leo (SGP)	Robert Edelman (USA)			Remy Teyssou (FRA)
	Walla Dempsey (USA)			Stephen Thomas
				Walla Dempsey
				Yee-Sin Leo

^a^Working group leader

Following the framework provided by the 2009 WHO classification, the experts were asked to select one of three scientific working groups according to their expertise (Table 1):

1. Clinical Endpoints working group, to develop endpoints for moderate and severe plasma leakage, bleeding, and organ involvement (heart, liver, CNS)
2. Endpoint validation working group to develop plan to validate endpoints; and
3. Dengue Illness Index working group, to develop a tool to characterize the disease experienced at the outpatient level.

Candidate clinical disease endpoints for interventional trials were developed by the working group and discussed during several teleconferences and two face-to-face meetings. The working groups reviewed the literature characterizing dengue disease severity as well as definitions established by international medical organizations, and proposed endpoints for each of the severity subcategories, including the principles of evaluation/validation of the proposed endpoints. The criteria used to develop the endpoints were: i) measurability, ii) reproducibility/robustness, iii) practicability/readiness for implementation, and iv) reference to the most recent scientific evidence. The group also agreed that the endpoint definitions should include a moderate severity level - on the causal path to severe disease – in addition to the severe disease endpoints.

Input on the draft endpoints was solicited from clinicians in dengue endemic regions during two regional dengue conferences (Workshop 2 at the 14th International Dengue Course, Cuba, August 2015; Workshop 3 at the Regional Conference of the International Research Consortium on Dengue Risk Assessment, Management, and Surveillance - IDAMS- regional Dengue conference, Vietnam, October 2015). During the second face-to-face consultation of the expert panel (Workshop 4, November 2015, see Fig. 1), the group agreed to employ the Delphi method to refine areas of agreement with respect to the three working groups [14]. The results of the Clinical Endpoints and Dengue Illness Index working groups are reported separately [15, 16].

### Delphi method

The Delphi method is a structured forecasting/decision-making tool that has successfully been applied for health research in many areas, including infectious diseases and global public health [17, 18]. The Delphi method creates conditions that are favorable to a convergence of opinions, while at the same time allowing moderators to clearly discern points of dissent. It usually takes the form of a written questionnaire and allows for anonymous and independent consultation and argumentation, thereby avoiding some of the drawbacks of face-to-face confrontations both on the social level (e.g. power relations within a group) and the practical level (time consuming, especially with geographically dispersed individuals) [19]. Responses are only visible to the moderator(s) and not to the participants in order to avoid self-moderation bias. The iterative nature of the consultation, building on feedback of the respondents, allows for the correction of potential bias in the initial questions, which is the main fragility of classical (non-iterative and non-interactive) queries. A potential problem with the Delphi method is the creation of a bottleneck towards convergence of opinions during the process [20, 21]. It is therefore important to use both open and closed questions, and to take into account the whole spectrum of opinions. The online platform used presented additional advantages such as low respondent drop-out rates (reducing effects of self-selection) and the possibility for respondents to revisit, complement or modify their answers during each round (generating more data). [22]

The Delphi query was subdivided into three panels: an Overall Approach (OA) Panel, a Clinical Endpoints Panel, and a Dengue Illness Index Panel (Fig. 1). Here we concentrate on the OA panel. The Delphi process was conducted online using the MESYDEL platform [23] developed by the Spiral Research Center at the University of Liège (Belgium). Three rounds of Delphi queries were launched and analyzed between May and August 2016. Questions were designed by the project organizers in close collaboration with the Spiral Research Center and were predominantly open-ended for the OA panel, allowing participants to raise additional elements, suggestions and opinions. Respondents were also encouraged to explain their choice for closed-ended and multiple choice questions. A subsequent round mainly focused on ambiguities or disagreement in the previous round, clarifying areas of disagreement and fostering more consensus on approaching the issue differently according to the participants’ input. A third round gauged participants’ appraisal of both the content and form of the Delphi.

The key topic areas covered in the OA panel of the consultation were:

1. Need for standardized clinical endpoints & operationalization of severe endpoints
2. Utility of moderate severity endpoints
3. Hospitalization as a moderate endpoint
4. Potential ramifications for public health practice
5. Granularity of endpoints
6. Validation of endpoints

Quantitative data for closed questions was tabulated using software analysis. Written responses were analyzed using a software ‘tagging’ approach. In this approach, the software selected parts of text from individual respondents and then grouped the text into function areas of a shared concern (although opinions might differ), a specific theme or subject that occurred regularly through various responses, or, on the contrary, a minority viewpoint that brought in new and different information. This approach resulted in a group of tags, indicating a set of relevant issues, for each question in each round of the Delphi. Grouped and tabulated under tags, the responses to each question were analyzed a second time to further develop the specific issues and check the pertinence of the tags themselves. The issues and themes thus identified served to elaborate new questions, or to modify existing questions in the next round of the Delphi. Particular attention was paid to areas of disagreement.

## Results

### Demographics and participation rates

Sixty-four individuals (including the 27 working group members), representing a total of 17 countries, were invited to the OA Panel. Thirty-nine of them (from 16 countries) agreed to participate (Fig. 1) and thirty-eight actively responded beginning with round 1 (Table 2). Respondents then self-selected to participate in either or both of the other two panels: the Clinical Endpoints Panel and the Dengue Illness Index Panel (Fig. 1).

**Table 2:** Delphi consensus approach – results of the overall approach round 1 and 2

Questions	Round 1 (*N* = 38)*				Round 2 (*N* = 28)*			
	Yes		No		Yes		No	
*Need for standardized clinical endpoints and operationalization*	No.	(%)	No.	(%)	No.	(%)	No.	(%)
1.1.1 Should specific clinical endpoints be defined for dengue interventional trials?	32	(84)	0	(0)				
1.1.2 If not, briefly explain why not.								
1.1.3 Do you think we also need clinical endpoint definitions for pathogenesis research?	31	(82)	1	(3)				
1.2.1 Do you think operationalization of severe dengue endpoints is necessary for interventional trials?	31	(82)	0	(0)				
2.1.1 Please let us know what information you consider essential to further characterize these endpoints.								
Ideal time to collect clinical samples and clinical information					26	(93)	2	(7)
Details on how to measure endpoints (specific assays, instrumentation, etc)					25	(89)	3	(11)
Chart with endpoints and checkboxes					18	(64)	10	(36)
Different instructions for children and adults					17	(61)	11	(39)
2.1.2 Could you please provide additional suggestions/ideas?								
*Moderate severity endpoints*								
1.3.1 Do you think there should be a moderate disease severity category?	29	(76)	3	(11)				
1.3.2 Briefly explain why or why not.								
2.2.1 The goal of this project is to develop endpoints for dengue intervention clinical research. These endpoints are meant for interventional clinical research only and we were never meant to modify or substitute the 2009 WHO classification. Do you think moderate endpoints should be proposed based on the best scientific evidence available so far and validated afterward utilizing ongoing or new studies?					27	(96)	1	(4)
2.2.2 Please explain your answer.								
*Hospitalization as moderate endpoint*								
1.4.1 Should prevention of hospitalization be the endpoints for dengue interventional trials?	14	(37)	18	(47)				
1.4.2 Briefly explain why or why not.								
2.4.1 Do you think that information on hospitalization should be collected during interventional clinical trials and used in conjunction with other endpoints of disease severity?					25	(89)	1	(4)
2.4.2 What is the main reason why information on hospitalization should be collected during an interventional trial?								
2.4.3 Please explain your answers.								
*Potential ramifications for public health practice*								
1.5.1 Do you think the severe/moderate dengue severity endpoints for research purposes should be based on/aligned with the dengue classification for severity as set forth WHO 2009?	22	(58)	10	(26)				
1.5.2 Do you think moderate disease severity endpoint definitions will impact public health practices?	19	(50)	12	(32)				
1.5.3 Briefly explain why or why not.								
2.3.1 Can you please elaborate on why do you think the severe/moderate endpoints should or should not be based on the 2009 WHO dengue classification?								
2.3.2 Can you suggest ways to minimize the potential impact of these endpoints for research on public health practices?								
*Granularity of endpoints*								
1.6.1 To measure disease severity in interventional research, do you think that a categorical system (e.g. moderate vs. severe) or a numerical point system (e.g. 1–10) would be better?	18	(47)	13	(34)				
1.6.2 Briefly explain your selection.								
2.5.1 Should a categorical system be developed first?					20	(71)	6	(21)
2.5.2 Do you think it’s a good idea to develop a numerical system in parallel and that relative weight / importance / comparability be assessed prospectively?					20	(71)	7	(25)
*Validation of endpoints*								
1.7.1 Should suggested endpoints be validated with large prospective data sets?	28	(74)	3	(8)				
1.7.2 If not, briefly explain why not.								

*Note: the total number of participants who agreed and disagreed to a specific question may not equal the column total of all active participants for that round because of non-responders, that is, participants were not obliged to respond to a specific question to proceed to the next question. The percentages in parentheses were calculated based on the number of active respondents in each round, not on the actual number of respondents for each individual question. For this reason, the percentages of ‘yes’ and ‘no’ for each question do not necessarily add up to 100%

The participants of the OA panel identified themselves as practicing in the following sectors: 50% from academia, 32% from industry, 26% from public health practice, 29% from clinical practice. These percentages add to more than 100%, as the respondents could select several options. The participation rate was 97% in the first round with a 74% completion rate of the questionnaire. In the second round, the participation rate was slightly lower with72%.

### Need for standardized clinical endpoints and operationalization

Round 1 of the Delphi OA panel indicated an agreement on the utility of clinical endpoints for interventional research (84%), for pathogenesis research (82%) and on the need to operationalize these endpoints (82%) (Table 2). In round 2, participants were asked which information they thought was essential to further characterize these endpoints. Information considered to be essential included: i) ideal time to collect clinical samples and information, (93% of the participants); ii) details on how to measure endpoints (e.g. specific assays, instrumentation; which was indicated by 89%; iii) chart with endpoints and checkboxes (64%); and iv) different instructions for children and adults (61%).

### Moderate severity endpoints

Seventy-six percent of the respondents agreed that a moderate disease category would be desirable as severe disease is considered rare and requires large sample sizes to measure during interventional clinical trials. In addition, defined moderate disease endpoints would allow a better characterization of the disease spectrum, and if properly defined, would indicate a stage in the progression of the disease and could have predictive value.

Although nearly all participants were in support of developing moderate endpoints, many pointed out the inherent challenges including the fact that they could not be statistically separated in a previous effort [6].

The respondents assigned different background rationales to the need of moderate disease severity endpoints, namely i) the importance of detecting small changes (as these changes may be incremental); ii) the rare occurrence of severe forms of dengue in clinical studies; and iii) the need to better characterize the disease spectrum of dengue. In addition, some respondents went beyond the research context mentioning iv) patient management and disease burden. In round 2, a majority of participants (96%) confirmed that moderate endpoints should be proposed based on the best scientific evidence available, which includes validation by ongoing or new studies. In addition, the need for prospective studies to validate the endpoints was spontaneously raised by some respondents.

### Hospitalization as moderate disease endpoint

In the first round, a slight majority (47% versus 37%) felt hospitalization would not be a meaningful endpoint for dengue interventional trials, primarily due to the heterogeneity of hospitalization polices among countries and regions, and between private and public hospitals within a country. Although the information hospitalization is relatively easy to ascertain (and likely be recorded regardless), the heterogeneity would still present a significant confounder for any direct comparisons.

However, 43% of respondents who elaborated on their response (see Table 2 question 1.4.2) argued that hospitalization could be one endpoint among others. It was also mentioned that despite the inherent heterogeneity, the hospitalization rate would still be a good proxy reflecting health expenditure. Yet other respondents implicitly questioned the pertinence of hospitalization as a measure for public health impact, next to its impracticality as a clinical endpoint according to the respondents. These opinions stand in contrast to those who think that hospitalization is an important endpoint from a public health perspective. In round 2, the majority of respondents agreed (89%) that data on hospitalization should be collected during interventional trials and used in conjunction with other endpoints of disease severity. When prompted to identify why such information should be collected, the main response categories were i) to measure the impact of an intervention on the health system (46%); ii) that hospitalization reflects disease severity and could be used as a proxy for moderate disease severity (35%).

In summary, hospitalization may be of limited use either to measure public health impact or as a proxy for disease severity, but questions around hospitalization stimulated numerous concerns. A conclusion that might garner approval by all is that hospitalization can bring additional information provided that a) it is used in conjunction with other variables; and b) it is carefully contextualized according to the time and place where it is collected.

### Potential ramifications for public health practice

Questions revolved around the alignment of severe/moderate dengue severity endpoints with the WHO 2009 dengue classification (Table 2, question 1.5.1) and around the concern that moderate disease severity endpoint definitions might in turn impact public health practices when implemented (1.5.2).The later question was accompanied by an open question (1.5.3) in which the participants were asked to elaborate on their answer.

In round 1, 58% were in favor of an alignment with the WHO guidelines. The second round included an open question (Table 2, question 2.3.1) asking the participants to explain in more detail. Fifty-seven percent of the respondents were in favor of an alignment with the WHO guidelines and pointed out that the WHO classification had clinical utility and that it was important to maintain a historical and practical continuity. Several also pointed out that the 2009 WHO classification was a good platform for further adaptation and refinement. The remaining respondents (*n* = 5) did not position themselves clearly, indicating that the feasibility of alignment with WHO remains to be seen in practice. When the respondents were analyzed for their sector of activity, nearly 82% from the public health sector and 70% from the clinical sector favored alignment with the current WHO classification.

The development of moderate endpoints generated the most divergent discussion with respect to possible ramifications for public health practice. About half of the participants (50%) thought that ramifications will impact public health practice (32% declined). Some respondents were unsure about the precise meaning of ‘public health practice,’ whether it referred to patient triage, treatment, or to hospitalization practices. This also revealed different visions about the objective and scope of the moderate disease endpoints: while for some respondents a moderate category would help to prioritize who should be hospitalized, others feared that adding moderate endpoints would engender confusion with respect to the current WHO guidelines. At the same time, many respondents thought that the moderate category will have no public health impact, irrespective of WHO alignment.

Round 2 concentrated on what should be done to minimize the public health impact of the research endpoints. Here two main groups of answers stood out. About 25% of the 19 participants who responded conceptualized this as a communication issue – highlighting the need to clearly explain the uses and objectives of these moderate severity endpoints. About 25% stated that the question was unclear or irrelevant. The comments also exemplified paradoxical expectations with respect to moderate disease endpoints: that while moderate classification was being developed for research purposes, there were concerns about possible public health practice consequences in terms of hospitalization and economic burden.

In summary, the main concern raised with respect to the public health ramifications was the possible confusion that might be created in disease endemic countries by classifications and definitions of severe disease currently used for clinical management versus new clinical endpoints developed for research purposes. Yet, other respondents were confident that local diagnosis and management guidelines were well established and would not be impacted by the dissemination of new research endpoints as long as the purpose of the new endpoints was clearly communicated.

### Granularity of endpoints

The degree of granularity to be achieved by the endpoints was a matter of discussion. When asked if the endpoints for clinical research should follow a categorical system (e.g. moderate vs. severe) or a numerical point system (e.g. 1–10), 47% preferred a categorical system and 34% preferred a numerical system. A categorical system was perceived as a first priority in the second round (71% in favor), with the option to develop a numerical system once more data are available (data on the relative weights/importance of the different manifestations in subcategories of severe/moderate disease). Reasons in favor of a categorical system included the ease in use and communication of such a system and its greater robustness with regard to heterogeneity. The experts felt that a numerical system implied a greater precision, which is currently not underpinned by the available data. In addition, a numerical system implied linearity of increasing severity, and that severity with the same number score is comparable between severity categories (i.e. between leakage and a given organ dysfunction).

Proponents of a numerical system liked the increased accuracy. With a numerical system, average severity could be compared between groups, and other features like duration of symptoms could easily be associated and analyzed as well. The argument also takes up the need for more granularity in the endpoints.

### Validation of endpoints

A majority (74%) agreed that the suggested endpoints should be validated using large prospective data sets. The way forward for validation of the endpoints was discussed in the structured expert consultation as well as in the Delphi. There was a broad agreement that the candidate clinical endpoints [15] should be validated using available data sets as well as prospectively collected data sets. Major criteria for validation of these endpoints are i) accuracy to reflect severe/moderate disease, ii) robustness in different environments, and iii) practical considerations.

### Satisfaction with Delphi

Ninety-four percent of the 17 active respondents expressed satisfaction that their input was taken into account and that the feedback provided throughout the process was sufficient and addressed all relevant issues. One comment reflected a desire for feedback to reflect the representativeness of the Delphi panels, while other comments expressed value in the Delphi exercise to map out certain contentious positions.

## Discussion

The results of the structured expert consultation and the Delphi approach clearly indicate support for the need of standardized clinical endpoints for interventional research and for the development of specific guidance on what data to collect, including the type of specimens, medical investigations, and even suggested time points for collection. A large majority of participants was also in favor of developing moderate endpoints, in spite of the fact that earlier attempts to quantify evidence-based moderate endpoints were not successful [6]. The slight majority of participants felt that these endpoints for research should be aligned with the 2009 WHO classification to leverage the existing body of knowledge and for continuity and consistency.

A slight majority of participants felt that hospitalization would not constitute a standalone endpoint for dengue interventional trials (except for its significance in resource expenditure), since the criteria for hospitalization vary widely within regions. However, most participants felt that hospitalization was a valuable piece of information that should be captured and considered together with other clinical outcomes to get a more complete picture of the effect of interventions on a patient outcome.

Some of the participants expressed concerns that the introduction of moderate endpoints for interventional research might have an impact on public health practice. A slight majority of respondents agreed with this position and pointed out that a moderate category might be adopted by physicians to discern between patients that require hospitalization or not. A slight minority was not concerned by possible impacts because the effort of endpoint development is clearly for interventional research trials use only and would simply not be feasible in routine (non-research) clinical settings in most dengue-endemic countries. The clinical endpoints generated here are designed to provide guidance for interventional clinical research only and are not meant to modify or substitute existing classification systems or influence clinical management decisions. A concerted effort will be made by the organizers of this project to clearly communicate that these endpoints are meant exclusively to facilitate interventional research studies.

During this Delphi approach, respondents naturally brought in matters of clinical management, disease burden and hospitalization when elaborating their vision on both moderate/severe disease research endpoints and the WHO 2009 guidelines. Thus, the disease categories/endpoints that this Delphi addressed were put into a relationship with clinical practice beyond the scope of research and interventional trials alone. Consistent with the purpose of this consultation, some participants (Table 2 question 1.5.1,) argued that a separation between the research context and the clinical context is important for the elaboration and use of the endpoints suggested by this Delphi.

Regarding granularity, a slight majority preferred a categorical system (e.g. moderate versus severe) to a numerical scoring system (e.g. 1–10) since it would be easier to use in interventional research trials, and the data currently available are not sufficiently robust to support a numerical system. As additional data on disease severity are collected, a numerical scoring system might be subsequently developed to improve precision in the measurement of clinical outcomes during a trial. A large majority agreed that the proposed endpoints should be validated using large prospective data sets.

The Delphi queried a broad segment of experts from various geographical regions with heterogeneous professional backgrounds related to dengue. As indicated earlier, the results do not allow correlating specific issues or opinions with the geographical background of the respondents. It should be noted, however, that some of the answers do suggest that the resources available in low-income countries may have an impact on the feasibility and comparability of the endpoints to be developed and other information (such as hospitalization) to be gathered. Political and economic factors, such as the role and presence of private hospitals with respect to public hospitals, may also influence how dengue is charted and severity is evaluated in certain geographical areas. Knowledge of the role of such factors, and a view on their distribution across geographical areas, will contribute to the effort of developing comparable standardized endpoints.

The next step in this process is to begin a validation process for the utility of the consensus endpoints. In the absence of a formal ‘gold standard’, the validation of accuracy is the most challenging task. In the past, the validation of accuracy has been carried out using medical interventions (e.g. ICU level monitoring and IV fluid therapy, blood transfusion, shock resuscitation) as reference categories [6]. The validation of the accuracy of moderate endpoints for dengue severity needs to take into account the causal path to the corresponding severe disease endpoint (e.g. fluid accumulation leading to severe vascular leakage).

The evaluation of robustness includes the frequency distribution of the abnormal variables included in the outcome definitions, stratified among other factors by geography and age (group). Large variability of the frequency distributions would indicate underlying factors that influence abnormal values and therefore the comparability of data between different settings. In addition, the repeatability of the measurements needs to be assessed, especially for variables that include a subjective component of assessment.

Practical considerations include the quality and availability of the variables required for the endpoint definitions in different data sets. Here we suggest distinguishing between ‘minimal data sets’ and optimal or full data sets for endpoint definitions. The minimal data sets would entail the most robust and readily available variables that are necessary for endpoint definition. Certain endpoints might not even be classifiable with a minimal data set (e.g. for severe/moderate cardiac involvement). The most important endpoints (e.g. the endpoints responsible for the majority of severe disease in dengue) should at least be reflected in the minimal data set.

In order to evaluate this strategy, we first suggest investigating existing prospectively collected data sets with regard to the candidate endpoints definitions. In addition to the categories mentioned above, this would also allow the identification of potential gaps in the data sets and lead towards clear requirements for future prospective data sets.

## Conclusions

The results of this iterative process indicated a clear need for standardized clinical endpoints for dengue interventional research. Challenges that were identified include: i) the complexity of developing moderate disease research endpoints for dengue; ii) the potential influence of clinical endpoints for interventional research on clinical practice; iii) communication of the purpose and proper use of these endpoints to clinical and public health officials; iv) the need to evaluate the suggested candidate endpoints with well-designed prospective studies.

For this project, experts from academia, industry and clinical practice from several dengue endemic countries were invited to address an important gap in vaccine and therapeutic research. Their continued efforts over 2 years resulted in the development of candidate clinical endpoints for moderate and severe dengue disease that are meant to facilitate the design of interventional clinical trials. While these endpoints will need to be validated with existing or future prospective data sets, this presents a significant step forward towards the standardization and harmonization of clinical trials for dengue interventions. The structured process, utilizing an expert panel and the Delphi method, proved useful for reaching agreement and documenting areas where further discussion is needed.

## Abbreviations

IDMC: Independent data monitoring committee
NIAID: National Institute of Allergy and Infectious Diseases
OA: Overall Approach
PDC: Partnership for Dengue Control
WHO: World Health Organization
